# Effects of Opioids, Benzodiazepines, Gabapentinoids, and Antidepressants on Fracture Risk in Alcohol‐Related Cirrhosis

**DOI:** 10.1111/liv.70667

**Published:** 2026-05-03

**Authors:** Frederik Kraglund, Thomas Deleuran, Marie Aarup Storgaard, Eva Sædder, Peter Jepsen

**Affiliations:** ^1^ Department of Hepatology and Gastroenterology Aarhus University Hospital Aarhus Denmark; ^2^ Department of Clinical Pharmacology Aarhus University Hospital Aarhus Denmark; ^3^ Department of Gastroenterology and Hepatology Aalborg University Hospital Aalborg Denmark; ^4^ Department of Biomedicine, Health Aarhus University Aarhus Denmark; ^5^ Department of Clinical Epidemiology Aarhus University Hospital Aarhus Denmark; ^6^ School of Medicine University of Nottingham Nottingham UK

**Keywords:** accidental falls, alcoholic, bones, cross‐over studies, Denmark, drug‐related side effects and adverse reactions, fractures, liver cirrhosis, registries

## Abstract

**Background:**

Patients with alcohol‐related liver cirrhosis (ALD cirrhosis) have an increased risk of falls and fractures. Opioids, benzodiazepines, gabapentinoids, and antidepressants are known to increase fall risk.

**Aims:**

We aimed to determine the effects of opioids, benzodiazepines, gabapentinoids, and antidepressants on fracture risk in ALD cirrhosis.

**Methods:**

Using Danish nationwide registries, we identified all patients diagnosed with ALD cirrhosis in 2000–2023 and their subsequent hospitalisations for fracture. Redeemed prescriptions defined exposure to opioids, benzodiazepines, gabapentinoids, and antidepressants. We applied Cox and Poisson regression to estimate fracture risks with and without exposure, adjusting for confounders. To minimise confounding, we also used a self‐controlled case‐crossover design.

**Results:**

Among 24 743 patients with newly diagnosed ALD cirrhosis, the 5‐year fracture risk was 19.0% (95% CI: 18.4%–19.4%). Fracture risk was increased during the first 30 days after initiation of opioids (adjusted hazard ratio [aHR]: 3.20, 95% CI: 2.30–4.45), benzodiazepines (aHR: 2.17, 95% CI: 1.37–3.76), or gabapentinoids (aHR: 2.43, 95% CI: 1.66–3.55), whereas antidepressant initiation did not affect fracture risk (aHR: 1.07, 95% CI: 0.62–1.84). Similar within‐person results were obtained using the self‐controlled design. Notably, the risk‐increasing effects of opioids, benzodiazepines, and gabapentinoids decreased with the duration of exposure, and none of the drugs increased fracture risk if the patient had been using them for more than 365 days.

**Conclusions:**

In ALD cirrhosis, fracture risk is increased when treatment with opioids, benzodiazepines, or gabapentinoids is initiated, but not with long‐term use. Precautionary measures are recommended when initiation of these drugs is indicated.

## Lay Summary

People with alcohol‐related liver cirrhosis have a high risk of fractures, especially shortly after starting certain medications. In this nationwide Danish study, starting opioids, benzodiazepines, or gabapentinoids was linked to a markedly higher fracture risk in the first month, while antidepressants were not. The increased risk declined over time and was not seen with long‐term use, suggesting extra caution is needed at first‐time prescription of the drugs.

## Key Points

- Patients with cirrhosis have high fracture risk and frequent exposure to fall‐risk–increasing drugs, including opioids, benzodiazepines, gabapentinoids, and antidepressants.
- Using nationwide data and a self‐controlled design, we evaluated fracture risk across patterns of use for these drug classes.
- Fracture risk was increased following initiation of opioids, benzodiazepines, and gabapentinoids.
- Continued use was associated with weaker risk increases across all four drug classes, whereas long‐term use was not associated with increased fracture risk.
- These findings support precautionary measures when initiating these drugs in patients with cirrhosis.

**Abbreviations**
aHR: adjusted hazard ratio
ALD: alcohol‐related liver disease
ATC‐code: Anatomical Therapeutic Chemical code
CNS: central nervous system
ICD‐10: International Classification of Diseases, 10th revision
IR: incidence rate
SSRI: selective serotonin reuptake inhibitor

## 1 Introduction

Liver cirrhosis increases the risk of falls and fractures through multiple mechanisms [1, 2, 3]: Cognitive dysfunction, particularly hepatic encephalopathy [4], and reduced bone mineral density are central [5]. Alcohol use not only exacerbates these mechanisms [2] but also acts as an independent risk factor for falls and fractures [6]. This is particularly important in cirrhosis due to alcohol‐related liver disease (ALD cirrhosis), where hazardous alcohol use is prevalent [7]. Furthermore, the prognosis of falls and fractures is significantly worse in cirrhosis [8, 9], underscoring the potential benefits of prevention.

Several prescription drugs have been associated with an increased risk of falls [10]. Among these, central nervous system (CNS)‐depressants such as opioids and benzodiazepines and CNS‐active drugs with sedative and psychomotor‐impairing effects such as gabapentinoids and antidepressants are of particular concern in ALD cirrhosis because they are strongly associated with falls in the general population [11, 12, 13], are increasingly prescribed [10], and are widely used in cirrhosis [14]. Furthermore, opioids, benzodiazepines, and antidepressants are metabolised in the liver, which may result in longer half‐lives, higher bioavailability, accumulation of active metabolites, and synergistic effects in ALD cirrhosis due to decreased hepatic metabolism, porto‐systemic shunting, and alcohol‐medication interactions [15, 16].

Despite their frequent use and potential for harm, the effects of opioids, gabapentinoids, and antidepressants on falls or fractures in cirrhosis have not been evaluated. Benzodiazepines have been linked to an increased risk of falls in cirrhosis [17], but this association may be overestimated since users often differ systematically from non‐users by underlying indications such as anxiety, agitation, and insomnia, which themselves increase the risk of falls.

We therefore investigated whether opioids, benzodiazepines, gabapentinoids, or antidepressants increase the risk of fractures in patients with ALD cirrhosis, using both cohort and self‐controlled designs to minimise confounding.

## 2 Methods

### 2.1 Setting

This nationwide, registry‐based study was conducted in the Danish population of 5 992 734 citizens (1 January 2025). In Denmark, all residents have free, tax‐supported access to general practitioners and public hospital care. The private healthcare sector in Denmark is very small [18], and cirrhosis is treated exclusively in the public sector. Prescription drugs are supported by a tax‐financed graduated reimbursement system where a citizen's annual expenditures cannot exceed 4735 DKK (~600 €) as of 2025 [19]. A unique identifier (the CPR‐number) issued to all Danish citizens at birth or immigration allows unambiguous individual‐level linkage across all the national healthcare registries, as well as complete follow‐up until death or emigration in the Civil Registration Registry. The National Patient Registry records data on all hospital contacts since 1995 [20], and the National Prescription Registry records all redeemed drug prescriptions since 1997 [21]. According to the Danish Data Protection Act, studies based on data from Danish healthcare registries do not require approval from an ethics committee or written consent.

### 2.2 Study Population

We included all patients diagnosed with ALD cirrhosis (International Classification of Diseases, 10th revision [ICD‐10]‐code: K70.3×) between 1 January 2000 and 31 March 2023 using the National Patient Registry. Baseline was the date of the first ALD cirrhosis diagnosis. Baseline cirrhotic decompensation was defined as any of the following diagnoses occurring before or at the time of the ALD cirrhosis diagnosis: diagnoses of ascites, variceal bleeding, spontaneous bacterial peritonitis, hepatorenal syndrome, paracentesis, or varices ligation. Baseline cancer, epilepsy, anxiety disorders, depression, diabetes, osteoporosis, ischaemic stroke or transient ischaemic attack, and cardiovascular disease were defined as any diagnosis or disease‐defining drug given before or at the time of the ALD cirrhosis diagnosis. Baseline hazardous alcohol use was defined as diagnoses implying hazardous alcohol use within 1 year before the ALD cirrhosis diagnosis, and prior fracture was defined as any bone fracture within 5 years before the ALD cirrhosis diagnosis. Because epilepsy was assumed to be associated with fractures and the propensity to use benzodiazepines and gabapentinoids, the cohort was restricted to patients without epilepsy diagnosed before baseline. All registry codes used to define the study population and covariates are listed in Table S1.

### 2.3 Exposures

Using the National Prescription Registry, we retrieved all prescriptions for opioids, benzodiazepines, gabapentinoids, and antidepressants redeemed by the study population before and after their first ALD cirrhosis diagnosis. We included prescriptions for tablets, capsules, suppositories, and transdermal opioid patches (collectively, “units”). By our definition, the exposure started on the date the prescription was redeemed.

We estimated the number of days' supply as the number of units dispensed divided by the number of units prescribed per day. When dosage instructions were missing, the prescribed daily units were imputed as the mean from prescriptions for the same product (identical brand, manufacturer, formulation, route, and strength). For prescriptions marked “as needed”, we assumed 1 unit/day. Overlapping prescriptions were concatenated into continuous exposure episodes. Surplus units from overlapping prescriptions were not carried forward.

We distinguished between treatment initiation, continued use, and long‐term use based on prescriptions redeemed both before and after the first ALD cirrhosis diagnosis. Treatment initiation was defined as the first 30 days of the first continuous episode in treatment‐naïve patients (or the full episode if it was ≤ 30 days). Continued use comprised the remainder of the first episode beyond day 30, as well as all subsequent episodes until the user became ‘long‐term’. Long‐term use was defined as use occurring in patients who had accumulated at least 365 days of prior exposure to the respective drug class. To assess heterogeneity within drug classes and aid clinical applicability, we additionally defined exposures at the level of individual drugs with more than 10 000 prescriptions during follow‐up. These are listed in Table S2. All Anatomical Therapeutic Chemical (ATC)‐codes used to define exposures are listed in Table S1.

### 2.4 Outcomes

We defined the outcome as any bone fracture (Table S1). Diagnosis codes given during outpatient visits were ignored. To account for delays in hospital coding, fracture dates were moved 1 day earlier than the admission date. If a fracture and a prescription were recorded on the same date after this adjustment, the fracture was assumed to have occurred first.

### 2.5 Study Designs

We aimed to estimate the absolute risk of fractures; the effects of treatment initiation (≤ 30 cumulative days of exposure), continued use (31–365 cumulative days of exposure), and long‐term use (> 365 cumulative days of exposure); and the within‐person effects of treatment initiation. To achieve this, we implemented two study designs: a traditional cohort design and a self‐controlled design.

#### 2.5.1 Cohort Design

Using the traditional cohort design, we estimated the absolute risk of fractures and the effect of treatment initiation, continued use, and long‐term use on the hazard rate of fracture. Patients were followed from the ALD cirrhosis diagnosis date until the first fracture, death, emigration, or 30 June 2025, whichever occurred first. We assumed that sex, age, calendar year, decompensation, cancer, anxiety disorders, depression, diabetes, osteoporosis, ischemic stroke or transient ischemic attack, cardiovascular disease, hazardous alcohol use, prior fracture, and use of multiple medications (polypharmacy) influenced both the propensity to use opioids, benzodiazepines, gabapentinoids, and antidepressants and the risk of fractures. Therefore, these variables were included as confounders. The exposure was time‐varying, meaning that patients were classified as exposed from the day they redeemed the prescription and for the full duration of exposure (as detailed above), and then they were unexposed until they redeemed a subsequent prescription. The drug exposure categories for each drug in the cohort study were no current use of the drug (reference), treatment initiation, continued use, and long‐term use.

#### 2.5.2 Self‐Controlled Case‐Crossover Design

Traditional cohort studies are susceptible to uncontrolled confounding, whereas self‐controlled studies are not [22]. Therefore, to assess whether unmeasured confounding influenced the results of the cohort study, we used a self‐controlled case‐crossover design in patients who experienced a fracture during follow‐up. The week leading up to the first fracture was designated as the ‘case period’, and the 1‐week periods leading up to 30 days before the fracture, 2 months before the fracture, and so on up to 11 months before the fracture were designated ‘control periods’ (Figure S1). Fractures less than 60 days after the ALD cirrhosis diagnosis were ignored because there would be no control periods for those fractures. The drug exposure categories in the case‐crossover design were opioid initiation, benzodiazepine initiation, gabapentinoid initiation, and antidepressant initiation, with the reference being ‘long‐term, continued, or no use’ of the given drug. Each case period and control period was categorised as ‘exposed’ if treatment initiation occurred during that period [23]. Since alcohol may be used to self‐medicate the same symptoms as the drugs of interest (pain, anxiety, insomnia, depression) and can increase fall and fracture risk, we included hazardous alcohol use as a time‐varying confounder. Case and control periods in which a patient received a diagnosis indicating hazardous alcohol use were categorised as alcohol exposed.

To investigate the effects in the older population, we conducted an analysis restricted to patients ≥ 60 years at baseline. To assess potential effect modification by liver disease severity, we performed subgroup analyses stratified by cirrhosis decompensation status at baseline. Finally, to assess effect heterogeneity within drug classes, we repeated the analyses at the level of individual drugs with more than 10 000 prescriptions during follow‐up (Table S2) and at least five observed fractures in the exposed group.

### 2.6 Statistics

#### 2.6.1 Cohort Analyses

In the cohort design, the cumulative incidence of fractures was estimated with death as a competing event. To study the effect of the drugs on the hazard rate of fractures, we used Cox proportional hazards regression clustered by patient to estimate adjusted hazard ratios (aHR), adjusted for baseline confounders and time‐varying use of either of the other studied drug classes. The exposure to the drugs was time‐varying, meaning that patients could switch between exposure levels during follow‐up, that is, it was an ‘as‐treated’ analysis [24]. We added an interaction term between sex and age, because we assumed that the hazard rate of fractures increases more steeply with age among women.

#### 2.6.2 Self‐Controlled Case‐Crossover Analyses

In the case‐crossover design, odds ratios (ORs) were computed using conditional logistic regression clustered by patient and adjusted for time‐varying hazardous alcohol use and treatment initiation of any of the other studied drug classes. Because fractures are rare outcomes, the ORs obtained from this analysis can be interpreted as relative risks [23]. In the subgroup analyses of individual drugs, time‐varying adjustment for the many other individual drugs could not be computed.

### 2.7 Sensitivity Analyses

We conducted sensitivity analyses to assess the robustness of our assumptions and analytical approach. First, because the actual medication use underlying an “as needed” prescription cannot be directly observed, we evaluated alternative assumptions in which such prescriptions corresponded to no use and to 0.5, 1 (main analysis), and 2 units per day in both study designs. Second, because vertebral compression fractures may occur spontaneously and initially present with nonspecific back pain, analgesics may be prescribed before the fracture is diagnosed, introducing potential reverse causation. We therefore conducted a sensitivity analysis excluding vertebral fractures and restricting outcomes to fractures more typically associated with trauma in both study designs. Third, to address potential confounding by acute clinical deterioration prompting drug initiation, we conducted a sensitivity analysis adjusting for recent hospitalisation. In the case‐crossover design, hospital admission within the preceding 30 days was included as a time‐varying covariate as a proxy for acute illness or clinical instability that could both trigger medication initiation and increase fracture risk.

### 2.8 Declaration of AI Technologies in the Writing Process

During the preparation of this work, the authors used ChatGPT 5 and Grammarly for language editing. After using this service, the authors reviewed and edited the content as needed and take full responsibility for the content of the publication.

## 3 Results

We included 24 743 patients with ALD cirrhosis diagnosed between 2000 and 2023. During 86 634 person‐years of follow‐up, 6023 patients experienced at least one fracture, while 15 853 died without a fracture. At the time of cirrhosis diagnosis, the median age was 60 years (interquartile range: 53–67), 69% were male, 39% had decompensated cirrhosis, 32% had hazardous alcohol use, and 23% had a prior fracture (Table 1).

**TABLE 1:** Baseline demographics at ALD cirrhosis diagnosis. Patients are stratified by use of opioids, benzodiazepines, gabapentinoids, or antidepressants at baseline.

	Non‐users	Opioid users	Benzodiazepine users	Gabapentinoid users	Antidepressant users	Total
*N*	17 895	2352	2425	525	3379	24 743
Male sex, *N* (%)	12 876 (72)	1557 (66)	1471 (61)	329 (63)	1938 (57)	17 092 (69)
Age at diagnosis, median (IQR)	60 (52–67)	61 (54–67)	57 (50–64)	60 (54–67)	60 (53–67)	60 (53–67)
Calendar year of diagnosis, median (IQR)	2011 ('05−'16)	2011 ('06−'16)	2007 ('04−'12)	2017 ('13−'20)	2012 ('08−'17)	2011 ('05–'16)
Cirrhotic decompensation, *N* (%)	7201 (40)	856 (36)	798 (33)	200 (38)	1186 (35)	9654 (39)
Cancer, *N* (%)	1939 (11)	410 (17)	243 (10)	96 (18)	385 (11)	2809 (11)
Anxiety and obsessive‐compulsive disorders, *N* (%)	184 (1)	63 (3)	97 (4)	34 (7)	157 (5)	437 (2)
Affective disorders with depression, *N* (%)	554 (3)	206 (9)	212 (9)	63 (12)	584 (17)	1325 (5)
Osteoporosis, *N* (%)	845 (5)	326 (14)	145 (6)	76 (15)	320 (10)	1477 (6)
Diabetes, *N* (%)	2912 (16)	651 (28)	377 (16)	191 (36)	776 (23)	4403 (18)
Stroke or transient ischemic attack, *N* (%)	711 (4)	139 (6)	95 (4)	44 (8)	238 (7)	1110 (5)
Hazardous alcohol use, *N* (%)	5403 (30)	707 (30)	974 (40)	143 (27)	1263 (37)	7848 (32)
Cardiovascular disease, *N* (%)	2517 (14)	510 (22)	336 (14)	125 (24)	595 (18)	3702 (15)
Prior fracture, *N* (%)	3572 (20)	829 (35)	679 (28)	173 (33)	979 (29)	5598 (23)
Polypharmacy (≥ 5 drugs), *N* (%)	4781 (27)	1783 (76)	1395 (58)	440 (84)	2251 (67)	9080 (37)
Polypharmacy (≥ 10 drugs), *N* (%)	619 (4)	668 (28)	369 (15)	184 (35)	695 (21)	1844 (8)

Compared with the full cohort, opioid and gabapentinoid users were more likely to have a prior cancer diagnosis, osteoporosis, diabetes, cardiovascular disease, or prior fracture. Benzodiazepine and antidepressant users were more likely to have hazardous alcohol use. Benzodiazepine users were younger at cirrhosis diagnosis and more often diagnosed in earlier calendar years, whereas gabapentinoid users were diagnosed in later years and more often had anxiety disorders. Antidepressant users were more often female and had a higher prevalence of depression. The prevalence of polypharmacy was markedly higher among users of any of the four drug classes—especially opioid and gabapentinoid users—compared with non‐users (Table 1). The number of prescriptions and the commonest non‐free‐text indications for each drug with ≥ 10 000 redeemed prescriptions in the study cohort are listed in Table S2.

### 3.1 Cohort Design

**FIGURE 1:**
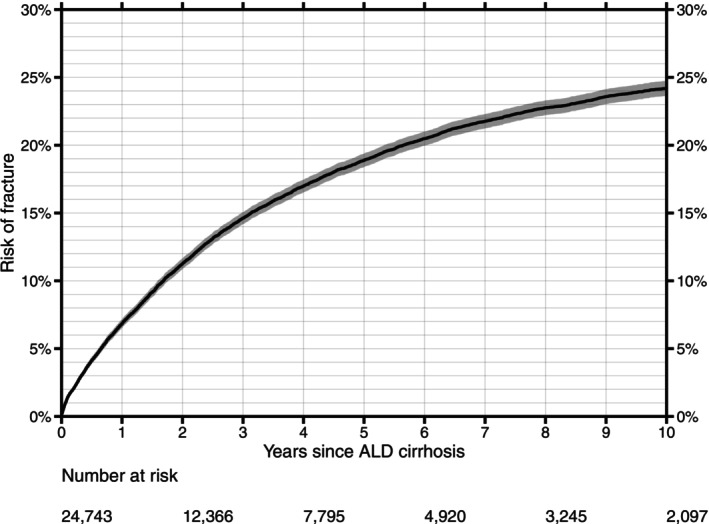
Cumulative incidence of fractures following the first ALD cirrhosis diagnosis. The grey area indicates the 95% confidence interval.

The risk of suffering at least one fracture was 6.8% (95% confidence interval [CI]: 6.5%–7.2%) after 1 year, 14.6% (95% CI: 14.1%–15.1%) after 3 years, 19.0% (95% CI: 18.4%–19.4%) after 5 years, and 24.2% (95% CI: 23.6%–24.7%) after 10 years (Figure 1). The overall incidence rate (IR) of fractures was 69.5 per 1000 person‐years (6023 fractures in total).

**FIGURE 2:**
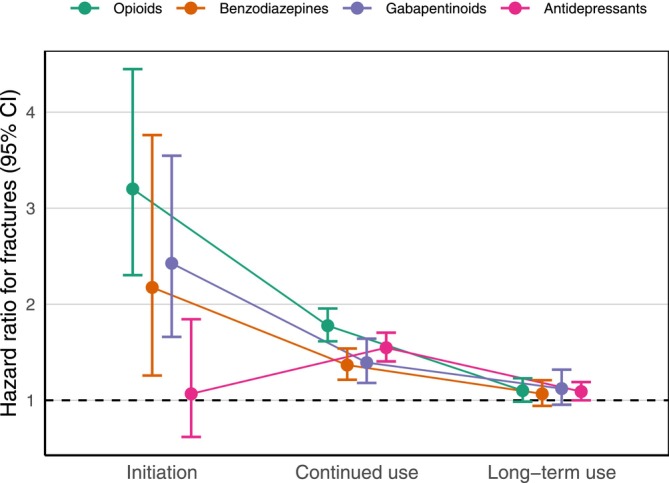
Hazard ratios from the cohort study comparing fracture risk during initiation, continued use, and long‐term use by drug class. Points indicate hazard ratio estimates and whiskers show 95% confidence intervals. The reference category is no current use.

Opioid initiation tripled the rate of fractures (aHR 3.20, 95% CI: 2.30–4.45), continued use almost doubled the rate (aHR 1.78, 95% CI: 1.61–1.96), while long‐term use did not significantly affect the rate (aHR 1.10, 95% CI: 0.98–1.23). This temporal risk pattern was also observed for benzodiazepines and gabapentinoids, whereas antidepressants stood out because there was no adverse effect of initiating them (Figure 2 and Table 2). Female sex, older age, hazardous alcohol use, osteoporosis, and prior fractures were associated with a higher fracture risk, and the risk of fractures increased more steeply with age among women (Table S3).

**TABLE 2:** Estimates from the cohort study (Cox regression) and the case‐crossover study (conditional logistic regression). In the cohort analysis, hazard ratios compare initiation, continued use, and long‐term use with no current use of the drug. In the case‐crossover analysis, odds ratios compare initiation with long‐term, continued, or no use of the drug.

	Cohort study aHR (95% CI)	Case‐crossover study, OR (95% CI)
Opioids (ref. no use)		
Treatment initiation	3.20 (2.30–4.45)	2.28 (1.55–3.36)
Continued use	1.78 (1.61–1.96)	
Long‐term use	1.10 (0.98–1.23)	
Benzodiazepines (ref. no use)		
Treatment initiation	2.17 (1.37–3.76)	2.14 (1.22–3.74)
Continued use	1.37 (1.21–1.54)	
Long‐term use	1.07 (0.94–1.21)	
Gabapentinoids (ref. no use)		
Treatment initiation	2.43 (1.66–3.55)	1.93 (1.26–2.94)
Continued use	1.39 (1.18–1.64)	
Long‐term use	1.12 (0.95–1.32)	
Antidepressants (ref. no use)		
Treatment initiation	1.07 (0.62–1.84)	1.03 (0.59–1.78)
Continued use	1.55 (1.40–1.70)	
Long‐term use	1.09 (1.00–1.19)	

### 3.2 Case‐Crossover Design

**FIGURE 3:**
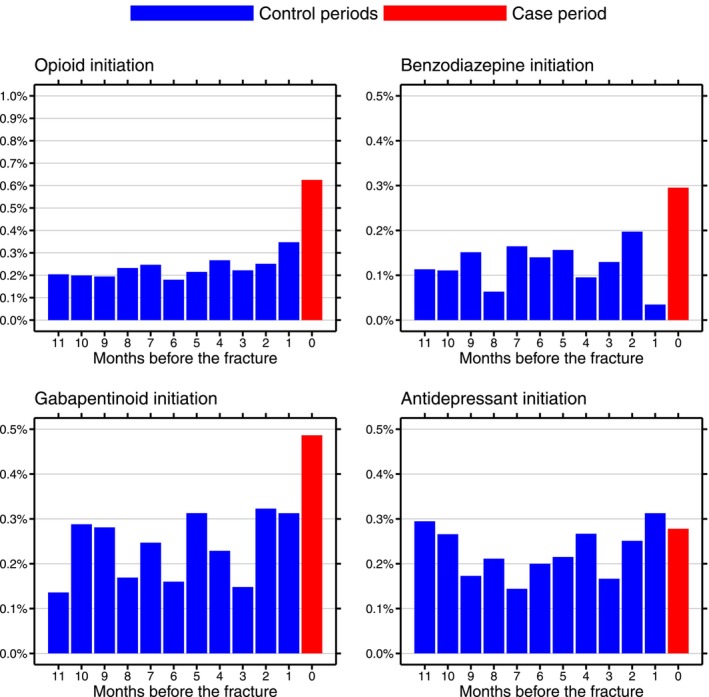
Probability of initiating opioids (top left), benzodiazepines (top right), gabapentinoids (bottom left), and antidepressants (bottom right) during the case and control periods in the case‐crossover study.

The self‐controlled design showed that a patient with ALD cirrhosis was markedly more likely to have initiated opioids shortly before having a fracture than they were at other times. The odds ratio was 2.29 (95% CI: 1.55–3.36), meaning that initiating opioids doubled the risk of suffering a fracture. For initiation of the three other drug classes, the OR was 2.25 (95% CI: 1.29–3.90) for benzodiazepines, 1.86 (95% CI: 1.21–2.85) for gabapentinoids, and 1.02 (95% CI: 0.59–1.76) for antidepressants (Figure 3 and Table 2).

For all four drug classes, the effects on fracture risk were more pronounced in patients ≥ 60 years with ORs of 2.44 (95% CI: 1.42–4.20) for opioids, 3.39 (95% CI: 1.43–8.04) for benzodiazepines, 2.82 (95% CI: 1.59–4.98) for gabapentinoids, and 1.44 (95% CI: 0.63–3.30) for antidepressants. Regarding cirrhosis severity, the effect was more pronounced in decompensated cirrhosis for benzodiazepines (OR 2.48, 95% CI: 1.06–5.76) and gabapentinoids (OR 2.32, 95% CI: 1.21–4.46), while it was less pronounced for opioids (OR 1.97, 95% CI: 1.03–3.78). For antidepressants, no association was found for both decompensated (OR 1.00, 95% CI: 0.39–2.54) and compensated cirrhosis (OR 1.05, 95% CI: 0.54–2.07).

**FIGURE 4:**
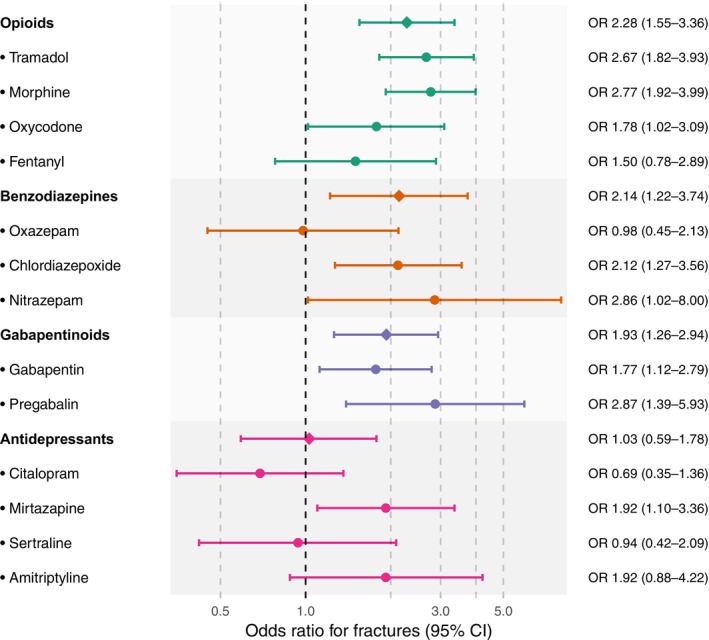
Odds ratios from the case‐crossover study for fracture risk during drug initiation by drug class and individual drug. Drugs with ≥ 10 000 redeemed prescriptions and ≥ 5 fractures during initiation are included. Points indicate odds ratio estimates and whiskers show 95% confidence intervals. The reference category is long‐term, continued, or no use.

At the individual drug level, patterns differed within classes. Among opioids, tramadol and morphine showed the strongest associations with fracture occurrence. Oxycodone was also positively associated with fractures, whereas transdermal fentanyl showed no clear association. For benzodiazepines, chlordiazepoxide and nitrazepam were positively associated with fractures, while oxazepam showed no association. Both gabapentin and pregabalin were associated with fractures. Among antidepressants, mirtazapine was associated with fractures, amitriptyline showed a non‐significant positive association, and neither citalopram nor sertraline showed any association (Figure 4).

### 3.3 Sensitivity Analyses

Overall, the results were robust across sensitivity analyses (Tables S4–S6). Alternative assumptions regarding medication use underlying “as needed” prescriptions did not meaningfully change the estimates. Restricting the outcome definition by excluding vertebral fractures resulted in slight attenuation of the opioid and gabapentinoid estimates, whereas the benzodiazepine estimates moved slightly further from the null. Adjustment for acute hospitalisation within the preceding 30 days slightly attenuated the estimates for opioids and benzodiazepines, while the gabapentinoid and antidepressant estimates remained essentially unchanged.

## 4 Discussion

In this nationwide registry study of patients with ALD cirrhosis, we found that initiation of opioids, benzodiazepines, and gabapentinoids markedly increased the risk of fractures, whereas antidepressant initiation showed no effect. Continued use (31–365 days) of all four drug classes was also associated with increased risk, though the magnitude was smaller than at initiation. In contrast, long‐term use (> 365 days) was not meaningfully associated with fracture risk for any of the drug classes. In stratified analyses, the association with treatment initiation was generally more pronounced in patients with decompensated cirrhosis for benzodiazepines and gabapentinoids, whereas the association for opioids was somewhat less pronounced. Our study makes several novel contributions: it clarifies the role of opioids, gabapentinoids, and antidepressants in fracture risk among patients with cirrhosis, and it distinguishes between the effects of treatment initiation, continued use, and long‐term use.

The observed effects of fall‐risk‐increasing drugs on fractures and, necessarily, falls are biologically plausible: CNS depression, sedation, dizziness, impaired balance, and orthostatic hypotension are well‐established mechanisms through which these drugs increase risk [10, 16]. For opioids, benzodiazepines, and gabapentinoids, the large effect observed at treatment initiation and attenuation over time is consistent with adverse effects that present immediately after initiation and may lessen as patients develop tolerance or adapt behaviorally [25, 26, 27]. The smaller or absent effects during continued and long‐term use may also reflect selection, as patients who do not tolerate treatment or experience adverse effects are more likely to discontinue therapy early, leaving a selected group of persistent users. The absence of an association in long‐term users should therefore be interpreted as a lack of observed excess risk in persistent users rather than as direct evidence of safety. We observed a different temporal pattern for antidepressants, with an increased risk with continued rather than initial use. Most of the prescribed antidepressants are selective serotonin reuptake inhibitors (SSRIs), for which adverse effects appear to develop more gradually [28]. Thus, the overall association for antidepressants might reflect delayed pharmacodynamic effects rather than immediate sedation. Tricyclic antidepressants and mirtazapine, by contrast, have strong antihistaminergic, anticholinergic, and α1‐adrenergic blocking properties that can cause more immediate sedation and orthostatic hypotension [29, 30], which may explain the clearer associations between fractures and initiation of these drugs.

Differences between individual drugs should nevertheless be interpreted with caution, as the study design and confounder adjustment set were optimised for estimating drug class effects. Treatment indication, disease severity, and clinical contexts such as palliative care likely play major roles in prescribing patterns and may confound the observed associations for individual drugs. With that caveat in mind, several pharmacokinetic and pharmacodynamic patterns are consistent with the data. In cirrhosis, reduced hepatocyte mass and porto‐systemic shunting impair hepatic clearance, increasing the bioavailability and toxicity risk of high‐clearance drugs [31]. Morphine and tramadol, both extensively metabolised in the liver, are strongly affected by cirrhosis, whereas oxycodone is less affected, and fentanyl, which bypasses first‐pass metabolism via transdermal patches, is least affected [31]. For gabapentinoids, both gabapentin and pregabalin are renally excreted, but pregabalin tends to cause more adverse effects [32]. Among benzodiazepines, short‐acting agents such as oxazepam and lorazepam are considered safer in cirrhosis [33], whereas nitrazepam has a substantially reduced clearance in ALD cirrhosis [34], and chlordiazepoxide accumulates through active metabolites in hepatic impairment [35]. All antidepressants are hepatically metabolised [36], which may further increase exposure in cirrhosis, but their pharmacodynamic profiles more likely determine their differences in terms of fracture risk.

Previous studies have established that patients with liver cirrhosis have an increased risk of both falls [1] and fractures [3] compared to population controls without cirrhosis. Tapper et al. recently reported 1‐ and 3‐year probabilities of “injurious falls” at 9.1% and 16.5% [1], similar to our estimates of 7.3% and 15.0%. Wester et al. estimated a much lower 10‐year fracture risk in ALD cirrhosis (13.5% vs. our 24.2%), but that study included patients diagnosed in 1969–1999, where the incidence of fractures was much lower than in 2000–2016 (35 vs. 60 per 1000 person‐years). The IR of 60 per 1000 person‐years in 2000–2016 in Sweden is comparable to our estimated IR of 69 per 1000 person‐years in 2000–2025 in Denmark [2], indicating strong agreement between Swedish and Danish data when aligned by calendar period.

Tapper et al. showed that benzodiazepines vastly increased the risk of falls in cirrhosis (adjusted OR 6.59, 95% CI: 3.76–11.59), consistent with our finding that benzodiazepine initiation doubled the risk of fractures. Given that 43% of patients with ALD cirrhosis continue to have hazardous alcohol use [7], the combined effects of alcohol and benzodiazepines likely amplify this risk. While the evidence that opioids, gabapentinoids, and antidepressants increase falls and fractures comes from studies of geriatric patients [10, 11, 12], this has not previously been examined in cirrhosis [17]. More recently, Tapper et al. reported that deprescribing benzodiazepines did not reduce falls and fractures, whereas discontinuing zolpidem (a non‐benzodiazepine hypnotic drug) did [37]. These results align with our finding that long‐term benzodiazepine use was not associated with excess fracture risk.

There are limitations to our study. First, the study relies on the validity of registry codes. In the National Patient Registry, the codes for ALD cirrhosis have been validated with a high positive predictive value [38], and codes for fractures generally have very high validity [39]. The National Prescription Registry used to quantify opioid, benzodiazepine, gabapentinoid, and antidepressant use provides highly valid data, as all drug sales are legally mandated to be recorded [40]. Second, it is practically impossible to determine exactly when patients used the dispensed drugs. Any misclassification of exposure is unlikely to depend on fracture risk and would therefore be non‐differential, biasing estimates toward the null. Misclassification is particularly relevant for “as needed” prescriptions, for which actual intake cannot be observed. Reassuringly, sensitivity analyses varying the assumed daily use for these prescriptions (0, 0.5, 1, and 2 units per day) did not materially change the results. Nevertheless, we were unable to evaluate dose–response relationships, and higher initial doses most likely confer greater fracture risk than lower doses. Third, some factors might affect both the risk of fractures and the propensity to use fall‐risk–increasing drugs. In the cohort analysis, we adjusted for variables assumed to influence both the exposure and the outcome at baseline: sex, age, and comorbidities, but unmeasured confounding like frailty might persist. Therefore, we also conducted a self‐controlled case‐crossover study to adjust for all confounding that was time‐invariant within a year, as well as time‐varying hazardous alcohol use. The results of the self‐controlled analyses were slightly closer to the null hypothesis across the board, indicating some residual confounding in the cohort analysis, but the overall accordance strongly supports a causal effect of opioid, benzodiazepine, and gabapentinoid initiation. To further address time‐varying confounding related to acute illness, we conducted a sensitivity analysis adjusting for hospitalisation within 30 days before the index date as a proxy for clinical deterioration. This adjustment only slightly attenuated estimates for opioids and benzodiazepines and had minimal impact on the other drug classes, supporting the robustness of the findings. Nevertheless, confounding by the clinical circumstances prompting treatment initiation cannot be fully excluded. Fourth, fracture subtypes are clinically heterogeneous. Vertebral fractures may occur spontaneously and can present with nonspecific back pain, potentially influencing diagnostic pathways. A sensitivity analysis excluding vertebral fractures resulted in only minor changes to the estimates, indicating that the main findings were robust. Finally, although analyses were stratified by decompensation status at baseline, liver function may change during follow‐up. Progression from compensated to decompensated cirrhosis may therefore have influenced patients' susceptibility to adverse drug effects.

Our findings suggest that fractures could be prevented by carefully considering the indication for initiation of the studied drugs. Importantly, the lack of a clinically meaningful effect of long‐term use across all four drug classes indicates that discontinuation in long‐term users is unlikely to reduce fracture risk. From a clinical standpoint, preventing initiation is often more feasible than achieving discontinuation [41]. The potential gains may be greatest in patients ≥ 60 years, where the fall‐risk‐increasing effects of these drugs were more pronounced, which is consistent with biological plausibility and prior studies [42, 43, 44, 45]. Preventing fractures is particularly important in cirrhosis, where surgical treatment is severely complicated and associated with excess morbidity, mortality, and resource use [8, 46, 47]. Patients with ALD cirrhosis and their physicians should therefore be aware of the fracture risks associated with initiating opioids, benzodiazepines, and gabapentinoids. Preventive measures include reconsidering the indication for initiation where possible, starting with lower or less frequent doses, and taking precautions to minimise falls at treatment initiation. At the same time, pain, anxiety, and depression are common in cirrhosis [48, 49], and the risk of self‐medication with alcohol is high. In such cases, prescribing may still be necessary despite the disadvantages. Precautionary choices may include oxazepam for anxiety or sedation and SSRIs for depression. Additional preventive strategies include exercise, home hazard reduction (e.g., removal of loose rugs), avoidance of polypharmacy, correction of vision, management of osteoporosis and orthostatic hypotension, and patient and caregiver information [50]. The generalizability of our findings should be considered in light of the Danish healthcare setting, which is characterised by universal coverage and prescribing practices that may differ from those in other regions. This is particularly relevant for opioids and benzodiazepines, where prescribing patterns vary substantially across countries. Consequently, the absolute risks and clinical implications of our findings may differ in settings with other prescribing cultures, healthcare access, and co‐prescribing patterns [51, 52].

In conclusion, initiation of opioids, benzodiazepines, and gabapentinoids substantially increased the risk of fractures in patients with ALD cirrhosis. This increase was not seen in long‐term users, but this should not be interpreted as direct evidence that long‐term use is safe.

## Author Contributions

All authors conceptualised the study and reviewed and edited the manuscript. F.K. and P.J. curated the data and conducted the formal analyses. F.K. wrote the original draft and created the data visualisations. P.J. acquired the funds to conduct the study and supervised the study.

## Funding

Peter Jepsen was supported by a grant from the Novo Nordisk Foundation (NNF18OC0054612). The funding organisation was not involved in the design, conduct, or decision to submit the manuscript for publication. All other authors report no financial support.

## Ethics Statement

According to the Danish Data Protection Act, studies based on data from Danish healthcare registries do not require approval from an ethics committee or written consent.

## Conflicts of Interest

The authors declare no conflicts of interest.

## Supporting information


**Figure S1:** Visualisation of the case‐crossover study design.
**Table S1:**. Registry codes used to define exposures, confounders, and outcomes.
**Table S2:**. Prescription information. The number of redeemed prescriptions and the commonest indications for each of the most prescribed drugs within opioids, benzodiazepines, gabapentinoids, and antidepressants in patients with ALD cirrhosis between 2000 and 2025.
**Table S3:**. The effects of the included confounders in the cohort study.
**Table S4:**. Sensitivity analyses using different assumptions about “as needed” prescriptions.
**Table S5:**. Sensitivity analysis excluding vertebral compression fractures.
**Table S6:**. Sensitivity analysis including acute hospitalisation within the last 30 days as a time‐varying confounder in the case‐crossover study.

## Data Availability

According to Danish law, national healthcare data cannot be shared publicly. Researchers may apply for data via https://sundhedsdatastyrelsen.dk/da/forskerservice.
