# Supplementary material for: Effects of Opioids, Benzodiazepines, Gabapentinoids, and Antidepressants on Fracture Risk in Alcohol‐Related Cirrhosis
Source: Liver Int. 2026 May 3;46:e70667. doi: 10.1111/liv.70667 (PMC13136785; doi:10.1111/liv.70667)
Supplement: Supplementary file 1 — Figure S1: Visualisation of the case‐crossover study design. Table S1:. Registry codes used to define exposures, confounders, and outcomes. Table S2:. Prescription information. The number of redeemed prescriptions and the commonest indications for each of the most prescribed drugs within opioids, benzodiazepines, gabapentinoids, and antidepressants in patients with ALD cirrhosis between 2000 and 2025. Table S3:. The effects of the included confounders in the cohort study. Table S4:. Sensitivity analyses using different assumptions about “as needed” prescriptions. Table S5:. Sensitivity analysis excluding vertebral compression fractures. Table S6:. Sensitivity analysis including acute hospitalisation within the last 30 days as a time‐varying confounder in the case‐crossover study. [file LIV-46-0-s001.docx]

**Supplementary material**

**Effects of opioids, benzodiazepines, gabapentinoids, and antidepressants on fracture risk in alcohol-related cirrhosis**

Frederik Kraglund**,** Thomas Deleuran**,** Marie Aarup Storgaard**,** Eva Sædder**,** Peter Jepsen

Table of contents

[Figure S1 2](#_Toc223727325)

[Table S1 3](#_Toc223727326)

[Table S2 5](#_Toc223727327)

[Table S3 6](#_Toc223727328)

[Table S4 7](#_Toc223727329)

[Table S5 9](#_Toc223727330)

[Table S6 10](#_Toc223727331)

Figure S1**. Visualization of the case-crossover study design.**


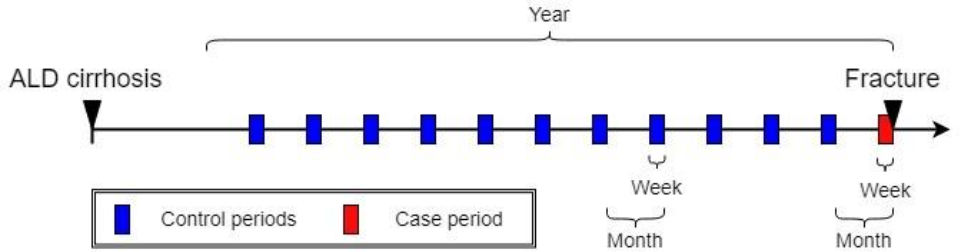


Table S1. **Registry codes used to define exposures, confounders, and outcomes.**

| **Variable** | **Definition** | **Registry codes** |
| --- | --- | --- |
| Cirrhosis due to alcohol-related liver disease |  | ICD-10: K70.3x |
| Cirrhosis decompensation |  |  |
|  | Ascites | ICD-10: R18x |
|  | Variceal bleeding | ICD-10: I85.0x, I86.4A |
|  | Spontaneous bacterial peritonitis | ICD-10: K65.8I |
|  | Hepatorenal syndrome | ICD-10: K76.7x |
|  | Paracentesis | NCSP: TJA10x |
|  | Treatment of esophagal varices | NCSP: JCA20, JCA22, JCA32 |
| Cancer |  | ICD-10: Cx |
| Epilepsy |  | ICD-10: G40x |
| Anxiety disorders |  |  |
|  | Phobic anxiety disorders | ICD-10: F40x |
|  | Other anxiety disorders | ICD-10: F41x |
|  | Obsessive-compulsive disorder | ICD-10: F42x |
| Depression |  |  |
|  | Depressive episode | ICD-10: F32x |
|  | Recurrent depressive disorder | ICD-10: F33x |
|  | Bipolar affective disorder, current episode depression | ICD-10: F31.3x-F31.5x |
|  | Dysthymia | ICD-10: F34.1x |
| Diabetes |  |  |
|  | Diabetes mellitus | ICD-10: E10x-E14x |
|  | Antidiabetics | ATC: A10x |
| Osteoporosis |  |  |
|  | Osteoporosis | ICD-10: M80x-M82x |
|  | Bisphosphonates | ATC: M05BAx |
|  | Bisphosphonates, combinations | ATC: M05BBx |
|  | Denosumab | ATC: M05BX04 |
| Ischemic stroke or transient ischemic attack |  |  |
|  | Ischemic stroke | ICD-10: I63x |
|  | Transient ischemic attack | ICD-10: G45x |
| Cardiovascular disease |  |  |
|  | Atrial fibrillation/flutter | ICD-10: I48x |
|  | Acute myocardial infarction | ICD-10: I21x |
|  | Old myocardial infarction | ICD-10: I25.2x |
|  | Heart failure | ICD-10: I50x |
|  | Atherosclerosis of arteries of extremities | ICD-10: I70.2x |
| Hazardous alcohol use |  |  |
|  | Acute alcohol intoxication | ICD-10: F10.0x |
|  | Harmful alcohol use | ICD-10: F10.1x |
|  | Alcohol dependence, active/episodic use | ICD-10: F10.24-F10.26 |
|  | Alcohol withdrawal | ICD-10: F10.3x-F10.4x |
|  | Alcohol-induced psychotic disorder | ICD-10: F10.5x |
| Opioids |  | ATC: N02Ax |
|  | Tramadol | ATC: N02AX02 |
|  | Morphine | ATC: N02AA01 |
|  | Oxycodone | ATC: N02AA05 |
|  | Fentanyl | ATC: N02AB03 |
|  | Ketobemidone with spasmolytic agent | ATC: N02AG02 |
|  | Codeine with paracetamol | ATC: N02AJ06 |
| Benzodiazepines |  | ATC: N05BAx, N05CDx |
|  | Oxazepam | ATC: N05BA04 |
|  | Diazepam | ATC: N05BA01 |
|  | Chlordiazepoxide | ATC: N05BA02 |
|  | Alprazolam | ATC: N05BA12 |
|  | Nitrazepam | ATC: N05CD02 |
| Gabapentinoids |  | ATC: N02BFx |
|  | Gabapentin | ATC: N02BF01 |
|  | Pregabalin | ATC: N02BF02 |
| Antidepressants |  | ATC: N06Ax |
|  | Citalopram | ATC: N06AB04 |
|  | Mirtazapine | ATC: N06AX11 |
|  | Sertraline | ATC: N06AB06 |
|  | Venlafaxine | ATC: N06AX16 |
|  | Amitriptyline | ATC: N06AA09 |
|  | Escitalopram | ATC: N06AB10 |
| Bone fractures |  | ICD-10: S12, S22, S32, S42, S52, S62, S72, S82, or S92 |

Table S2. **Prescription information.** The number of redeemed prescriptions and the commonest indications for each of the most prescribed drugs within opioids, benzodiazepines, gabapentinoids, and antidepressants in patients with ALD cirrhosis between 2000 and 2025.

|  | **Generic name** | **Number of prescriptions** | **Commonest indications (% of non-free-text)** |
| --- | --- | --- | --- |
| **Opioids** | | **282,209** | **Pain (99.6%)** |
|  | Tramadol | 98,536 | Pain (99.7%) |
|  | Morphine | 76,063 | Pain (99.2%) |
|  | Oxycodone | 55,412 | Pain (99.9%) |
|  | Fentanyl | 15,651 | Pain (99.4%) |
|  | Ketobemidone with spasmolytic agent | 11,253 | Pain (98.8%), nausea/vomiting (1.3%) |
|  | Codeine with paracetamol | 10,435 | Pain (99.2%) |
| **Benzodiazepines** | | **139,292** | **Sedation (73.1%), insomnia (20.6%), anxiety (5.2%)** |
|  | Oxazepam | 41,493 | Sedation (91.9%), anxiety (7.9%) |
|  | Diazepam | 30,874 | Sedation (92.9%), anxiety (4.3%), seizures/cramps (2.2%) |
|  | Chlordiazepoxide | 19,088 | Sedation (93.9%), anxiety (4.3%), withdrawal (1.6%) |
|  | Alprazolam | 16,314 | Sedation (88.8%), anxiety (11.1%) |
|  | Nitrazepam | 11,326 | Insomnia (100%) |
| **Gabapentinoids** | | **71,205** | **Pain (82.8%), anxiety (12.6%), seizures/cramps (4.2%)** |
|  | Gabapentin | 44,435 | Pain (93.9%), seizures/cramps (5.7%), |
|  | Pregabalin | 26,770 | Pain (64.3%), anxiety (33.7%), seizures/cramps (1.6%) |
| **Antidepressants** | | **192,290** | **Depression (77.9%), anxiety (19.1%), pain (2.3%)** |
|  | Citalopram | 53,866 | Depression (82.8%), anxiety/sedation (16.9%) |
|  | Mirtazapine | 44,241 | Depression (87.4%), anxiety/sedation (12.1%) |
|  | Sertraline | 20,600 | Depression (71.6%), anxiety/sedation (26.6%) |
|  | Venlafaxine | 17,571 | Depression (80.9%), anxiety/sedation (18.7%) |
|  | Amitriptyline | 11,999 | Depression (39.3%), anxiety/sedation (35.8%), pain (24.5%) |
|  | Escitalopram | 10,218 | Depression (81.8%), anxiety/sedation (17.8%) |

Table S3**. The effects of the included confounders in the cohort study.**

|  |  | **aHR (95% CI)** |
| --- | --- | --- |
| Polypharmacy (ref. <5 drugs) | |  |
|  | 5-9 drugs | 1.01 (0.95−1.07) |
|  | ≥10 drugs | 1.03 (0.92−1.16) |
| Hazardous alcohol use | | 1.26 (1.19−1.33) |
| Male sex | | 0.80 (0.76−0.84) |
| Age (10-year increase) | | 1.06 (1.01−1.11) |
| Interaction: male sex and 10-year age increase | | 0.92 (0.87−0.97) |
| Calendar year (10-year increase) | | 0.98 (0.94−1.03) |
| Cirrhotic decompensation | | 1.06 (1.00−1.11) |
| Prior cancer | | 0.98 (0.89−1.08) |
| Anxiety disorder | | 0.99 (0.83−1.18) |
| Depression | | 1.03 (0.92−1.15) |
| Diabetes | | 1.03 (0.96−1.11) |
| Osteoporosis | | 1.35 (1.21−1.50) |
| Prior stroke or transient ischemic attack | | 1.08 (0.95−1.23) |
| Cardiovascular disease | | 1.02 (0.94−1.11) |
| Prior fracture | | 1.85 (1.74−1.96) |

Table S4**. Sensitivity analyses using different assumptions about “as needed”** **prescriptions.**

|  |  | Cohort study aHR (95% CI) | Case-crossover study, OR (95% CI) |
| --- | --- | --- | --- |
| **Sensitivity analysis (“as needed” prescriptions excluded)** | | | |
|  | Opioid initiation | 3.14 (2.25−4.38) | 2.30 (1.56−3.38) |
|  | Continued opioid use | 1.79 (1.62−1.97) |  |
|  | Long-term opioid use | 1.12 (1.00−1.25) |  |
|  | Benzodiazepine initiation | 2.18 (1.26−3.77) | 2.17 (1.24−3.80) |
|  | Continued benzodiazepine use | 1.35 (1.20−1.52) |  |
|  | Long-term benzodiazepine use | 1.06 (0.93−1.20) |  |
|  | Gabapentinoid initiation | 2.44 (1.67−3.57) | 1.86 (1.21−2.85) |
|  | Continued gabapentinoid use | 1.43 (1.21−1.68) |  |
|  | Long-term gabapentinoid use | 1.10 (0.93−1.29) |  |
| **Sensitivity analysis (“as needed” = 0.5 unit per day)** | | | |
|  | Opioid initiation | 3.12 (2.24−4.36) | 2.19 (1.48−3.24) |
|  | Continued opioid use | 1.79 (1.63−1.97) |  |
|  | Long-term opioid use | 1.11 (1.00−1.24) |  |
|  | Benzodiazepine initiation | 2.18 (1.26−3.77) | 2.17 (1.24−3.80) |
|  | Continued benzodiazepine use | 1.35 (1.20−1.52) |  |
|  | Long-term benzodiazepine use | 1.06 (0.94−1.21) |  |
|  | Gabapentinoid initiation | 2.44 (1.67−3.56) | 1.86 (1.21−2.85) |
|  | Continued gabapentinoid use | 1.43 (1.22−1.68) |  |
|  | Long-term gabapentinoid use | 1.08 (0.92−1.27) |  |
| **Main analysis (“as needed” = 1 unit per day** | | | |
|  | Opioid initiation | 3.20 (2.30−4.45) | 2.28 (1.55−3.36) |
|  | Continued opioid use | 1.78 (1.61−1.96) |  |
|  | Long-term opioid use | 1.10 (0.98−1.23) |  |
|  | Benzodiazepine initiation | 2.17 (1.37−3.76) | 2.14 (1.22−3.74) |
|  | Continued benzodiazepine use | 1.37 (1.21−1.54) |  |
|  | Long-term benzodiazepine use | 1.07 (0.94−1.21) |  |
|  | Gabapentinoid initiation | 2.43 (1.66−3.55) | 1.93 (1.26−2.94) |
|  | Continued gabapentinoid use | 1.39 (1.18−1.64) |  |
|  | Long-term gabapentinoid use | 1.12 (0.95−1.32) |  |
| **Sensitivity analysis (“as needed” = 2 units per day)** | | | |
|  | Opioid initiation | 3.21 (2.31−4.47) | 2.28 (1.55−3.36) |
|  | Continued opioid use | 1.78 (1.61−1.96) |  |
|  | Long-term opioid use | 1.11 (0.99−1.24) |  |
|  | Benzodiazepine initiation | 2.17 (1.26−3.76) | 2.17 (1.24−3.80) |
|  | Continued benzodiazepine use | 1.35 (1.20−1.52) |  |
|  | Long-term benzodiazepine use | 1.06 (0.94−1.21) |  |
|  | Gabapentinoid initiation | 2.43 (1.66−3.56) | 1.86 (1.21−2.85) |
|  | Continued gabapentinoid use | 1.43 (1.22−1.68) |  |
|  | Long-term gabapentinoid use | 1.09 (0.93−1.28) |  |

Table S5**. Sensitivity analysis excluding vertebral compression fractures.**

|  |  | Cohort study aHR (95% CI) | Case-crossover study, OR (95% CI) |
| --- | --- | --- | --- |
| Opioids (ref. no use) | |  |  |
|  | Treatment initiation | 2.75 (1.92−3.95) | 2.23 (1.48−3.36) |
|  | Continued use | 1.67 (1.52−1.85) |  |
|  | Long-term use | 1.10 (0.99−1.23) |  |
| Benzodiazepines (ref. no use) | | | |
|  | Treatment initiation | 2.30 (1.33−3.97) | 2.12 (1.17−3.82) |
|  | Continued use | 1.38 (1.22−1.56) |  |
|  | Long-term use | 1.06 (0.93−1.20) |  |
| Gabapentinoids (ref. no use) | | | |
|  | Treatment initiation | 2.07 (1.36−3.14) | 1.71 (1.09−2.67) |
|  | Continued use | 1.40 (1.19−1.66) |  |
|  | Long-term use | 1.13 (0.96−1.33) |  |
| Antidepressants (ref. no use) | | | |
|  | Treatment initiation | 1.12 (0.65−1.94) | 0.96 (0.55−1.70) |
|  | Continued use | 1.58 (1.43−1.74) |  |
|  | Long-term use | 1.09 (1.00−1.19) |  |

Table S6**. Sensitivity analysis including acute hospitalization within the last 30 days as a time-varying confounder in the case-crossover study.**

|  | Case-crossover study, OR (95% CI) |
| --- | --- |
| Opioid initiation | 2.09 (1.41−3.09) |
| Benzodiazepine initiation | 2.04 (1.16−3.59) |
| Gabapentinoid initiation | 1.96 (1.29−3.00) |
| Antidepressant initiation | 1.04 (0.60−1.80) |
